# Nonlinear Blind Compensation for Array Signal Processing Application

**DOI:** 10.3390/s18041286

**Published:** 2018-04-22

**Authors:** Jialu Huang, Hong Ma, Jiang Jin, Hua Zhang

**Affiliations:** School of Electronic Information and Communications, Huazhong University of Science & Technology, 1037 Luoyu Road, Wuhan 430074, China; huangjialu1987@126.com (J.H.); mahong@hust.edu.cn (H.M.); jinjiang@hust.edu.cn (J.J.)

**Keywords:** nonlinear blind compensation algorithm, array receiver, two-dimensional direction-of-arrival, spurious-free dynamic range, array signal processing

## Abstract

Recently, nonlinear blind compensation technique has attracted growing attention in array signal processing application. However, due to the nonlinear distortion stemming from array receiver which consists of multi-channel radio frequency (RF) front-ends, it is too difficult to estimate the parameters of array signal accurately. A novel nonlinear blind compensation algorithm aims at the nonlinearity mitigation of array receiver and its spurious-free dynamic range (SFDR) improvement, which will be more precise to estimate the parameters of target signals such as their two-dimensional directions of arrival (2-D DOAs). Herein, the suggested method is designed as follows: the nonlinear model parameters of any channel of RF front-end are extracted to synchronously compensate the nonlinear distortion of the entire receiver. Furthermore, a verification experiment on the array signal from a uniform circular array (UCA) is adopted to testify the validity of our approach. The real-world experimental results show that the SFDR of the receiver is enhanced, leading to a significant improvement of the 2-D DOAs estimation performance for weak target signals. And these results demonstrate that our nonlinear blind compensation algorithm is effective to estimate the parameters of weak array signal in concomitance with strong jammers.

## 1. Introduction

During the past few decades, array signal processing (ASP) has been a world-wide hot spot of research [1,2], with discoveries as diverse in application as wireless communications, multiple-input multiple-output (MIMO) radar and sonar [3,4,5,6]. ASP is concerned with the problem of extracting high dimensional information of interest which is received from an array of spatially distributed sensors and transmitted to a multi-channel array receiver. There are normally three units in an array system: antenna arrays, array receiver and array signal processing unit [7]. Moreover, an array receiver, comprising of multi-channel radio frequency (RF) front-ends, contains many nonlinear circuits, bringing about the vast majority of nonlinear distortion components in an array signal processing system.

Currently, much research regarding array signal processing focuses on the development of parameter estimation methods [8,9,10,11]. Amongst them, two-dimensional direction-of-arrival (2-D DOA) estimation is the hottest topic and most of the published literature focuses on parametric algorithms to deal with it [9,10,11]. These methods are proven to perform well in source direction finding under ideal settings, which ignore the negative effect of the nonlinear distortion components of an array signal processing system on source localization accuracy. However, due to the nonlinearity of an array receiver, the signal-to-interference-plus-noise ratio (SINR) of several target signals is reduced. Such imperfect methods lead to sharply deteriorated performance under a condition of low SINR. Therefore, further research is required to better address the parameters estimation problem of an array signal in such a scenario as an electromagnetic environment in which an array signal processing system works. 

Some effective methods [12,13] for such a scenario adopt high-order statistics to improve the direction-finding performance of array signal which, however, increase the computational complexity enormously compared with the methods such as the multiple signal classification (MUSIC) algorithm applying second-order statistics. To resolve the difficulty without increasing the computation burdens of the direction finding, the authors enhance the spurious-free dynamic range (SFDR) of an array receiver, which leads to significant SINR improvements of weak target signals. Therefore, to promote the development of array signal processing, an array receiver with high SFDR is a vital undertaking. Fewer researches for blindly mitigating the nonlinear distortion of multi-channel array receiving systems have been conducted, to the best of the authors’ knowledge. 

The nonlinearity mitigation technique for a one-channel receiving system has been reported in some papers [14,15,16,17,18,19,20,21,22,23]. Several preliminary ideas of Reference [14] offer some insight for this research. This paper proposes a nonlinear blind compensation method to eliminate the distortion product of band-limited signal by suppressing the out-of-band distorted signal. However, this method is only applicable to compensating memoryless and monotonic nonlinearity under the premise of the known input signal bandwidth. A digital post-compensation technique is adopted to mitigate the nonlinear distortion of the multicarrier receiving system [15]. Moreover, for linearizing a direct-conversion receiver (DCR), a full-band adaptive interference cancellation (FB-AIC) method has been proposed [16]. This method utilizes the out-band distortions to identify and extract the nonlinear model coefficients. Furthermore, in Reference [17], some effective works have been done to degrade the third-order intermodulation distortion of DCR severely. Recently, the paper [18] developed a blind nonlinear post-distortion algorithm with follow-up blind finite impulse response (FIR) equalizers to compensate for the distortion product in the concurrent dual-band receiver, the memory effects and the non-ideal wireless channel response. Although References [15,16,17,18,19] mainly focus on cancelling the third-order inter-modulation distortion, they fail to be applied to a wideband nonlinear system. The current authors’ initial research [20] proposed that minimizing the short-time energy of the nonlinearity in the frequency domain is regarded as the blind identification criterion. However, the identification strategy might have difficultly to coping with the situation where multiple unknown signals with different power levels exist simultaneously, due to the non-ideal frequency response characteristic of FIR. Moreover, in References [18,20,21,22,23], the nonlinear behavior model parameters are identified and extracted in the frequency domain, resulting in heavy computational costs.

To solve these identification problems, separating large signals (strong array signals) and small signals (nonlinear distortion components and weak array signals) in time domain according to the power level is appropriate. Large signals produce most of the nonlinear components and are usually of far higher power level compared with small signals. Thereupon, the instantaneous power residual error between the small signals and nonlinear model of large signals can be considered as an objective function of nonlinear model kernels. Consequently, a blind identification criterion is designed to adaptively resize nonlinear model coefficients and an improved weighed iterative method (IWI) is presented to minimize the instantaneous power residual error based on the method in research [24].

In References [25,26,27,28,29], some blind signal separation algorithms have been investigated; for instance, the singular value decomposition (SVD)-based and the eigenvalue decomposition (EVD)-based methods. The SVD-based algorithm is the standard due to its good performance and applicability, as confirmed in References [26,28,29]. As learned from the References [26,28,29], the performance for large signal extraction is mainly determined by an adequate choice of the number of the largest singular values, r. However, it is hard to precisely determine r on account of the unknown array signal. A smaller r will bring about a part of the large signals, while a bigger one will draw some unwanted nonlinearity or weak array signals into the large signals. Both the conditions will result in incorrect extracted outcomes. Furthermore, the engineering implementation of a SVD-based algorithm needs massive computational loads [29].

A novel nonlinear blind compensation algorithm is suggested to cope with array signal processing. Under the existing semiconductor process levels and device performance status, this study focuses on significantly improving the SFDR of an array receiver and the parameters estimation accuracy of the weak target signals, (for example, their 2-D DOAs). To the best of the authors’ knowledge, the MUSIC algorithm is one of the most widely-used high-resolution 2-D DOA estimation approaches. Therefore, the authors consider it to be an efficient azimuth-elevation angles estimation method for an array signal.

Additionally, the authors propose a novel blind signal separation algorithm, a spectrum reduction algorithm based on time-frequency conversion (SRA-TFC) algorithms wherein large signals and small signals are extracted based on the power level in the frequency domain. To follow, their time domain waveforms are obtained by the Inverse Discrete Fourier transform (IDFT) technique. Comparing with the traditional SVD-based method, the proposed method is only necessary to set the power threshold rather than the number of the largest singular values of constant change. Therefore, the method is highly suitable to blindly separate signals in the electromagnetic environment.

Moreover, the identification criterion should be applied to each channel for extracting its identification coefficients if each channel is regarded to be an independent and irrelevant nonlinear system. The disadvantages are as follows: first, the system hardware resource is increased significantly. Most importantly, the inconsistent compensation performances of different channels, especially the phase disturbance of the post-compensation array signal, are caused by the iterative computation error of the proposed identification criterion. However, the input array signal and the linear/nonlinear transfer functions of each channel are similar. A more suitable method is to blindly identify and extract the nonlinear model kernel coefficients of any one of the channels and then to employ the extracted kernel coefficients to synchronously mitigate the nonlinearity of all channels. Consequently, the authors propose an effective digital compensation method for an array signal processing system.

The arrangement of this paper is as follows: Section 2 describes the nonlinear blind compensation processing architecture for an array system and the theoretical demonstration of the proposed algorithm. It is followed up in Section 3 with the nonlinear blind mitigation architecture. The experimental results are given in Section 4 to demonstrate the performance improvement of the proposed algorithm. Finally, a short conclusion is drawn in Section 5.

## 2. System Model and Problem Analysis

### 2.1. ASP Architecture Based on Nonlinear Blind Compensation

**Figure 1:**
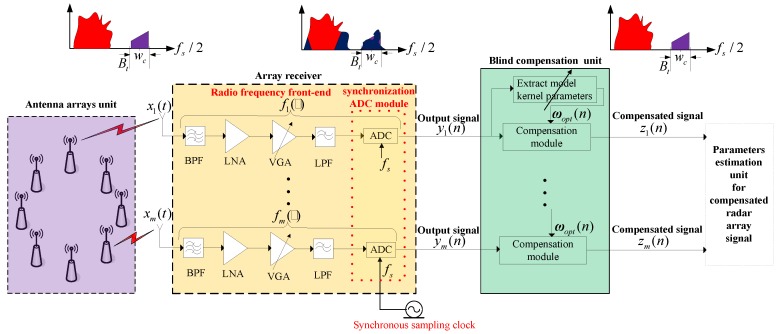
Block diagram of the nonlinear blind compensation processing for the array system.

Figure 1 shows a structural diagram of the nonlinear blind compensation processing for an array system, which is composed of an antenna arrays unit, array receiver and blind compensation unit. Where, $x_{i}(t)$, $y_{i}(n)$ are the analog input, digital output in the ith channel RF front-end, respectively; $z_{i}(n)$ is the ith channel post-compensation signal; $f_{s}$ means the sampling rate of the synchronous clock; $w_{c}$, $B_{t}$ stands for the center frequency and bandwidth of the target signal.

Additionally, in the sub-figure across the top of Figure 1, it contains three power spectrum schematic diagrams of different signals which are input signals, output signals and completely compensated signals of an array receiver, separately. All red regions in these diagrams mean the power spectrum of a large array signal, while all purple regions are that of weak target signals. Moreover, the blue region shows the power spectrum of the nonlinear distortion stemming from the array signal through the array receiver.

The antenna arrays unit exists at the head of the system, where it is to capture the array signal. Then, the array data is transmitted to the array receiver. Each channel RF front-end of the array receiver has some nonlinear analog circuits and an ADC (analog to digital converter). The analog module usually consists of a bandpass filter (BPF) with high suppression ratio, a low-noise amplifier (LNA), a variable-gain amplifier (VGA), a low-pass filter (LPF) and more. The full-band array signal is achieved by filtering the input signal with a BPF in each channel of the array receiver. It is then amplified to the required level by a first-stage LNA and a first-stage VGA. Additionally, the amplified signal without out-band clutter obtained by an LPF is transmitted to the synchronization ADC module in which all ADCs’ sampling clocks are from the same crystal oscillator. These procedures are applied to synchronously gather and output the array signal. The blind compensation unit is designed to mitigate or even eliminate the distortion product supplied from the multi-channel array processing system with the aim to improve the parametric estimation accuracy of the array signal. 

### 2.2. Theoretical Analysis of the Proposed Method

This section offers the feasibility analysis of the novel nonlinear blind compensation method which adaptively extracts the nonlinear model kernel coefficients of any one of the channels to mitigate the nonlinearity of all channels synchronously.

The bandwidth of the multi-channel nonlinear systems, tens of MHz, is much bigger than that of the target signals, several KHz. Thereby a same memoryless nonlinear model representing for every single channel of the nonlinear systems could validate the presented nonlinear compensation method [30]. Additionally, it is well known that the pth order orthogonal polynomials with completely uncorrelated terms contributes to only the pth order nonlinear distortion term. Thus, the orthogonal polynomials modeling for nonlinear systems can offer an intuitive means of compensation improvement analysis to every nonlinear term. The authors adopt the Chebyshev polynomials, one of the most frequently used orthogonal polynomials.

It is generally accepted that the second-order and third-order nonlinear terms are the key factors to exacerbate the SFDR performance of the nonlinear system [20]. Therefore, a Chebyshev nonlinear model with third-order non-linear terms could be modeled to approximate the multi-channel nonlinear systems. Therefore, the authors consider the ith channel RF front-end a discrete-time third–order Chebyshev orthogonal nonlinear system:

(1) $$\begin{array}{ccc} y_{i} & = & f_{i}(x_{i})\\ & = & a_{i}T_{0}(x_{i})+b_{i}T_{1}(x_{i})+c_{i}T_{2}(x_{i})+d_{i}T_{3}(x_{i}) \end{array}$$

where $f_{i}(•)$ represents the nonlinear transformation function of the ith channel RF front-end; $T_{p}(•)$ denotes pth order Chebyshev node; $x_{i}$ and $y_{i}$ are the simplified forms of $x_{i}(t)$ and $y_{i}(n)$, respectively; $a_{i}$ means the coefficient of the direct-current (DC) component; $b_{i}$ represents the linear term parameter, $c_{i}$ and $d_{i}$ are the coefficients of the second-order and third-order nonlinear terms.

The first three Chebyshev orthonormal polynomials are shown as:

(2) $$\begin{array}{l} T_{0}(x)=1\\ T_{1}(x)=x\\ T_{2}(x)=2x^{2}-1\\ T_{3}(x)=4x^{3}-3x \end{array}$$

substituting Equation (2) into Equation (1) gives:

(3) $$\begin{array}{ccc} y_{i} & = & a_{i}+b_{i}x_{i}+c_{i}(2x_{i}^{2}-1)+d_{i}(4x_{i}^{3}-3x_{i})\\ & = & (a_{i}-c_{i})+(b_{i}-3d_{i})x_{i}+2c_{i}x_{i}^{2}+4d_{i}x_{i}^{3} \end{array}$$

Equation (3) has its DC component assumed to be zero. Furthermore, the coefficient of linear term ($b_{i}$) is much bigger than that of nonlinear terms ($c_{i}$, $d_{i}$) in a weak nonlinear system. Thus, Equation (3) can be rewritten:

(4) $$\begin{array}{ccc} y_{i} & = & (b_{i}-3d_{i})x_{i}+2c_{i}x_{i}^{2}+4d_{i}x_{i}^{3}\\ & \approx & b_{i}x_{i}+2c_{i}x_{i}^{2}+4d_{i}x_{i}^{3}\;(a_{i}=c_{i}) \end{array}$$

The model parameters of any one of the channels are extracted, in this paper the first channel for an example. Via the blind compensation for the first channel nonlinear system, its inverse behavior model of the nonlinear system is obtained approximately [20]. Therefore, according to the knowledge of Reference [31], the inverse nonlinear behavior model can be defined as:

(5) $$\begin{array}{ccc} z_{i} & = & g_{1}(y_{i})\\ & = & \rho_{0}T_{0}(y_{i})+\rho_{1}T_{1}(y_{i})+\rho_{2}T_{2}(y_{i})+\rho_{3}T_{3}(y_{i}) \end{array}$$

where, $z_{i}(n)$ is reduced to $z_{i}$; $g_{1}(•)$ is the inverse of $f_{1}(•)$ (the nonlinear transformation function of the first channel) and its coefficient vector is represented by $g={\left[\rho_{0},\rho_{1},\rho_{2},\rho_{3}\right]}^{T}$; T denotes transposition operation.

Corresponding to Equation (4), the vector of power series coefficients of the first channel F is obtained:

(6) $$F={\left[0,b_{1},2c_{1},4d_{1}\right]}^{T}$$

The *p*-fold convolution vectors are calculated by:

$$F^{\left(p\right)}(p=0,1,2,3)$$

and the matrix H assembled by juxtaposing these column vectors as shown below:

(7) $$H=[F^{\left(0\right)},F^{\left(1\right)},F^{\left(2\right)},F^{\left(3\right)}]$$

Normally, for inversion, the dimensions of H is truncated to a 4 × 4 matrix, denoted as:

(8) $$H_{4\times 4}=\left[\begin{array}{cccc} 1 & 0 & 0 & 0\\ 0 & b_{1} & 0 & 0\\ 0 & 2c_{1} & {\left(b_{1}\right)}^{2} & 0\\ 0 & 4d_{1} & 4b_{1}c_{1} & {\left(b_{1}\right)}^{3} \end{array}\right]$$

Furthermore, Chebyshev series-power series conversion matrix T is truncated as the following:

(9) $$T=\left[\begin{array}{cccc} 1 & 0 & -1 & 0\\ 0 & 1 & 0 & -3\\ 0 & 0 & 2 & 0\\ 0 & 0 & 0 & 4 \end{array}\right]$$

g is gained by choosing from the second column of matrix $\Gamma =T\cdot (H^{-1})\cdot (T^{-1})$ as described in Reference [32]:

(10) $$\begin{array}{l} \rho_{0}=\frac{c_{1}}{b_{1}^{3}}\\ \rho_{1}=\frac{b_{1}^{4}-3b_{1}d_{1}+6c_{1}^{2}}{b_{1}^{5}}\\ \rho_{2}=-\frac{c_{1}}{b_{1}^{3}}\\ \rho_{3}=\frac{2c_{1}^{2}-b_{1}d_{1}}{b_{1}^{5}} \end{array}$$

Substituting Equation (10) into Equation (5) gives:

(11) $$z_{i}=\frac{c_{1}}{b_{1}^{3}}T_{0}(y_{i})+\frac{b_{1}^{4}-3b_{1}d_{1}+6c_{1}^{2}}{b_{1}^{5}}T_{1}(y_{i})-\frac{c_{1}}{b_{1}^{3}}T_{2}(y_{i})+\frac{2c_{1}^{2}-b_{1}d_{1}}{b_{1}^{5}}T_{3}(y_{i})$$

According to the knowledge of Chebyshev polynomials:

(12) $$\begin{array}{l} T_{0}(x)=1\\ T_{1}(x)=x\\ T_{n}(x)\times T_{m}(x)=\frac{T_{n+m}(x)+T_{n-m}(x)}{2}\;n\geq m \end{array}$$

Therefore, the result is:

(13) $$\begin{array}{ccc} T_{0}(y_{i}) & = & 1\\ T_{1}(y_{i}) & = & y_{i}=c_{i}+b_{i}T_{1}(x_{i})+c_{i}T_{2}(x_{i})+d_{i}T_{3}(x_{i})\\ T_{2}(y_{i}) & = & 2{\left(y_{i}\right)}^{2}-1\\ & = & 2{\left[(c_{i}+b_{i}T_{1}(x_{i})+c_{i}T_{2}(x_{i})+d_{i}T_{3}(x_{i})\right]}^{2}-1\\ & = & 2[c_{i}^{2}+b_{i}^{2}{\left(T_{1}(x_{i})\right)}^{2}+c_{i}^{2}{\left(T_{2}(x_{i})\right)}^{2}+d_{i}^{2}{\left(T_{3}(x_{i})\right)}^{2}\\ & & +2b_{i}c_{i}T_{1}(x_{i})+2c_{i}^{2}T_{2}(x_{i})+2c_{i}d_{i}T_{3}(x_{i})+2b_{i}c_{i}T_{1}(x_{i})T_{2}(x_{i})\\ & & +2b_{i}d_{i}T_{1}(x_{i})T_{3}(x_{i})+2c_{i}d_{i}T_{2}(x_{i})T_{3}(x_{i})]-1\\ T_{3}(y_{i}) & = & 4{\left(y_{i}\right)}^{3}-3y_{i}=y_{i}[2(2{\left(y_{i}\right)}^{2}-1)-1)=T_{1}(y_{i})[2T_{2}(y_{i})-1] \end{array}$$

This process is just concerned about linear term and lower nonlinear terms (the second-order and third-order ones).

Since:

$$|b_{i}|>>|d_{i}|,\;\left|b_{i}|>>|c_{i}\right|,$$

Thereby, it can be obtained:

(14) $$T_{1}(y_{i})\approx b_{i}T_{1}(x_{i})+c_{i}T_{2}(x_{i})+d_{i}T_{3}(x_{i})$$

(15) $$\begin{array}{ccc} T_{2}(y_{i}) & \approx & b_{i}^{2}T_{2}(x_{i})+4b_{i}c_{i}T_{1}(x_{i})+4c_{i}^{2}T_{2}(x_{i})+4c_{i}d_{i}T_{3}(x_{i})\\ & & +2b_{i}c_{i}(T_{1}(x_{i})+T_{3}(x_{i}))+2b_{i}d_{i}T_{2}(x_{i})+2c_{i}d_{i}T_{1}(x_{i})\\ & = & \left(6b_{i}c_{i}+2c_{i}d_{i})T_{1}(x_{i})+(b_{i}^{2}+4c_{i}^{2}+2b_{i}d_{i})T_{2}(x_{i})+(4c_{i}d_{i}+2b_{i}c_{i})T_{3}(x_{i}\right)\\ & \approx & 6b_{i}c_{i}T_{1}(x_{i})+b_{i}^{2}T_{2}(x_{i})+2b_{i}c_{i}T_{3}(x_{i}) \end{array}$$

(16) $$\begin{array}{ccc} T_{3}(y_{i}) & \approx & \left[c_{i}+b_{i}T_{1}(x_{i})+c_{i}T_{2}(x_{i})+d_{i}T_{3}(x_{i})][(12b_{i}c_{i}+4c_{i}d_{i})T_{1}(x_{i}\right)\\ & & +(2b_{i}^{2}+8c_{i}^{2}+4b_{i}d_{i})T_{2}(x_{i})+(8c_{i}d_{i}+4b_{i}c_{i})T_{3}(x_{i})\\ & & +2b_{i}d_{i}T_{4}(x_{i})+2c_{i}d_{i}T_{5}(x_{i})+6c_{i}^{2}+2b_{i}^{2}+2d_{i}^{2}-3]\\ & \approx & \left[c_{i}+b_{i}T_{1}(x_{i})+c_{i}T_{2}(x_{i})+d_{i}T_{3}(x_{i})](12b_{i}c_{i}T_{1}(x_{i})+2b_{i}^{2}T_{2}(x_{i})+4b_{i}c_{i}T_{3}(x_{i}\right)\\ & & +2b_{i}d_{i}T_{4}(x_{i})+2c_{i}d_{i}T_{5}(x_{i})+2b_{i}^{2}-3)\\ & \approx & 12b_{i}c_{i}^{2}T_{1}(x_{i})+2b_{i}^{2}c_{i}T_{2}(x_{i})+4b_{i}c_{i}^{2}T_{3}(x_{i})+6b_{i}^{2}c_{i}T_{2}(x_{i})+b_{i}^{3}T_{1}(x_{i})+b_{i}^{3}T_{3}(x_{i})\\ & & +2b_{i}^{2}c_{i}T_{2}(x_{i})+b_{i}^{2}d_{i}T_{3}(x_{i})+(2b_{i}^{2}-3)b_{i}T_{1}(x_{i})+6b_{i}c_{i}^{2}T_{1}(x_{i})+6b_{i}c_{i}^{2}T_{3}(x_{i})\\ & & +2b_{i}c_{i}^{2}T_{1}(x_{i})+b_{i}c_{i}d_{i}T_{2}(x_{i})+c_{i}^{2}d_{i}T_{3}(x_{i})+(2b_{i}^{2}-3)c_{i}T_{2}(x_{i})+6b_{i}c_{i}d_{i}T_{2}(x_{i})\\ & & +b_{i}^{2}d_{i}T_{1}(x_{i})+b_{i}d_{i}^{2}T_{1}(x_{i})+c_{i}d_{i}^{2}T_{2}(x_{i})+(2b_{i}^{2}-3)d_{i}T_{3}(x_{i})\\ & \approx & \left(2b_{i}^{3}-3b_{i})T_{1}(x_{i})+(12b_{i}^{2}c_{i}-3c_{i})T_{2}(x_{i})+(b_{i}^{3}-3d_{i})T_{3}(x_{i}\right) \end{array}$$

Substituting Equations (14)–(16) into Equation (11), the approximate value of the post-compensation signal $z_{i}$ is expressed as:

(17) $$\begin{array}{ccc} z_{i} & \approx & \frac{b_{1}^{4}-3b_{1}d_{1}}{b_{1}^{5}}(b_{i}T_{1}(x_{i})+c_{i}T_{2}(x_{i})+d_{i}T_{3}(x_{i}))\\ & & -\frac{b_{1}^{2}c_{1}}{b_{1}^{5}}(6b_{i}c_{i}T_{1}(x_{i})+b_{i}^{2}T_{2}(x_{i})+2b_{i}c_{i}T_{3}(x_{i}))\\ & & -\frac{b_{1}d_{1}}{b_{1}^{5}}[(2b_{i}^{3}-3b_{i})T_{1}(x_{i})+(12b_{i}^{2}c_{i}-3c_{i})T_{2}(x_{i})+(b_{i}^{3}-3d_{i})T_{3}(x_{i})]\\ & \approx & \frac{b_{i}}{b_{1}}T_{1}(x_{i})+\frac{b_{1}^{2}c_{i}-b_{i}^{2}c_{1}}{b_{1}^{3}}T_{2}(x_{i})+\frac{b_{1}^{3}d_{i}-b_{i}^{3}d_{1}}{b_{1}^{4}}T_{3}(x_{i}) \end{array}$$

Normally, the approximate value of $b_{1}$ is 1. Thereby, Equation (18) could be represented as:

(18) $$z_{i}\approx b_{i}T_{1}(x_{i})+\frac{b_{1}^{2}c_{i}-b_{i}^{2}c_{1}}{b_{1}^{3}}T_{2}(x_{i})+\frac{b_{1}^{3}d_{i}-b_{i}^{3}d_{1}}{b_{1}^{4}}T_{3}(x_{i})$$

Equation (18) allows for the conclusion that the nonlinear compensation process just suppresses the distortion product without affecting linear components which contain the weak signals.

It can be known from Equations (1) and (17) that the uncompensated amplitudes of the second-order and third-order terms are $c_{i}$ and $d_{i}$, respectively in the *i*th channel; while the post-compensation amplitudes of the second-order and third-order terms are $\frac{b_{1}^{2}c_{i}-b_{i}^{2}c_{1}}{b_{1}^{3}}$ and $\frac{b_{1}^{3}d_{i}-b_{i}^{3}d_{1}}{b_{1}^{4}}$, respectively. Therefore, the improvement degrees of the second-order and third-order nonlinear distortion components in the ith channel, $I_{i2}$ and $I_{i3}$ are obtained:

(19) $$\begin{array}{cc} I_{i2} & =10\mathrm{lg}\frac{{\left(c_{i}\right)}^{2}}{{\left(\frac{b_{1}^{2}c_{i}-b_{i}^{2}c_{1}}{b_{1}^{3}}\right)}^{2}}=20\mathrm{lg}\left|\frac{c_{i}}{\frac{b_{1}^{2}c_{i}-b_{i}^{2}c_{1}}{b_{1}^{3}}}\right|\\ & =20\mathrm{lg}\left|\frac{b_{1}^{3}c_{i}}{b_{1}^{2}c_{i}-b_{i}^{2}c_{1}}\right|\\ I_{i3} & =10\mathrm{lg}\frac{{\left(d_{i}\right)}^{2}}{{\left(\frac{b_{1}^{3}d_{i}-b_{i}^{3}d_{1}}{b_{1}^{4}}\right)}^{2}}=20\mathrm{lg}\left|\frac{d_{i}}{\frac{b_{1}^{3}d_{i}-b_{i}^{3}d_{1}}{b_{1}^{4}}}\right|\\ & =20\mathrm{lg}\left|\frac{b_{1}^{4}d_{i}}{b_{1}^{3}d_{i}-b_{i}^{3}d_{1}}\right| \end{array}$$

where, “lg” represents Naperian logarithm in base 10.

$G_{i}$ is defined as the *i*th channel’s gain. $PE_{i}$ means the difference between $G_{i}$ and $G_{1}$ (the first channel’s gain) and it is represented by:

(20) $$PE_{i}=G_{i}-G_{1}$$

and:

(21) $$\begin{array}{l} G_{i1}=20\;\mathrm{lg}\left|\frac{b_{i}}{b_{1}}\right|=PE_{i}\\ G_{i2}=20\;\mathrm{lg}\left|\frac{c_{i}}{c_{1}}\right|\\ G_{i3}=20\;\mathrm{lg}\left|\frac{d_{i}}{d_{1}}\right| \end{array}$$

where, $G_{i1}$, $G_{i2}$ and $G_{i3}$ are the linear, second-order and third-order components of $PE_{i}$, respectively.

The authors make a group of experiments on the values of $PE_{i}$ under different signal frequencies. The experimental results are from a widely-used 16-channel array receiver which is also applied to the nonlinear blind compensation processing of the array system. Among the values of $PE_{i}$ for each frequency point, for statistical convenience, only the maximum and minimum absolute values are listed in Table 1 where the absolute values of $PE_{i}$ range from approximately 0 to near 1. Consequently, in the real array receiving system, the reasonable estimation that $G_{i1}$ ranges from −1 dB to 1 dB can be obtained. Moreover, as shown in Table 1, most of the measurements are much smaller than 1. Therefore, for computational convenience, $G_{i2}$ and $G_{i3}$ both ranging from −1 dB to 1 dB is rational.

**Figure 2:**
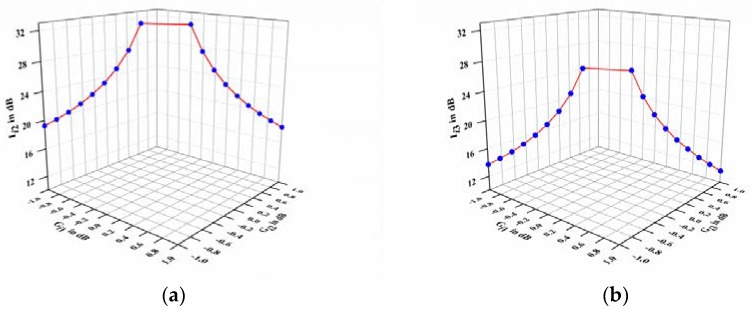
Computational results in the *i*th channel: (**a**) the improvement degrees of the second-order nonlinear term (*I_i_*_2_); (**b**) the improvement degrees of the third-order nonlinear term (*I_i_*_3_).

Based on Equations (19) and (21), the authors obtain the calculation results for $I_{i2}$ and $I_{i3}$, as shown in Figure 2, which demonstrates that $I_{i2}$ is approximately 16–32 dB and $I_{i2}$ is about 12–24 dB. Therefore, the results prove that this novel method could significantly compensate each channel’s nonlinear distortion of array receiver.

## 3. Proposed Mitigation Architecture for Array Receiver

It is the principle of the proposed algorithm that large signals create significant distortion product. The objective function is written as the minimal residual sum of squares between small signals and the nonlinear model of large signals.

This paper proposes, first, the blind compensation architecture in detail followed by the proposed IWI method for calculating nonlinear model parameters. Second, a SRA-TFC algorithm is given to separate time-domain large and small signals. Finally, the authors describe the comparisons to traditional SVD-based algorithms regarding the compensation performance and the computational complexity.

### 3.1. Nonlinear Blind Mitigation Structure

**Figure 3:**
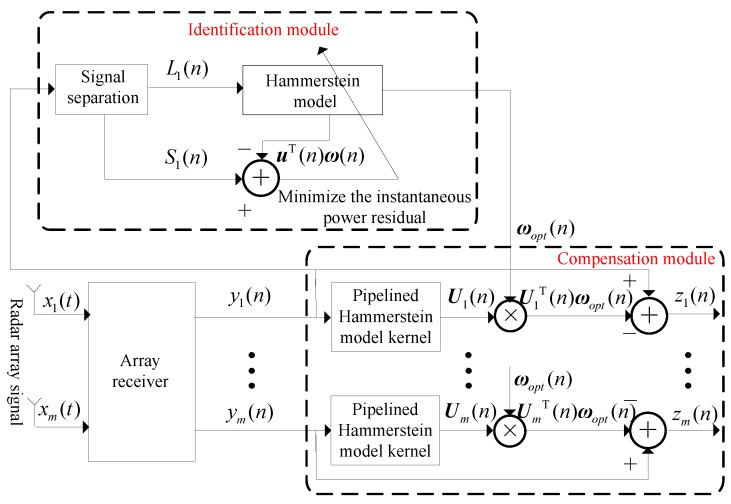
Proposed nonlinear blind mitigation structure for the array receiver.

Figure 3 demonstrates the proposed structure implemented in the blind compensation unit is made up of two basic modules of identification and compensation. The model parameters of the first-channel are extracted adaptively.

To dramatically improve the SFDR of the multi-channel array receiver, the nonlinear behavior model of the first channel RF front-end can be approximately expressed by the Hammerstein model, which is a cascade of static nonlinearity blocks followed by linear dynamical blocks [33]. The relationship between input and output can be expressed as:

(22) $$v(n)=\sum_{d=1}^{D}\sum_{r_{d}=0}^{N_{d}-1}h(r_{d},d){L_{1}}^{d}(n-r_{d})$$

where v(n) is the output signals of the discrete Hammerstein model; $L_{1}(n)$ means large signals in the first channel; D expresses the maximum value of the nonlinear order d (1≤d≤D); $N_{d}$ represents the memory depth of the dth order Hammerstein kernel; $h(r_{d},d)$ is the model kernel coefficient of the dth order with memory depth $r_{d}$; the total number of the Hammerstein kernel is:

(23) $$P=\sum_{d=1}^{D}N_{d}$$

Furthermore, the major function of the identification module is to generate identification parameters of a nonlinear model according to the objective function minimizing the power of N points error signal e(n) where:

$\omega (n)={\left[h(0,1)\;h(1,1)\;\ldots \;h(N_{1}-1,1)\;h(0,2)\;h(1,2)\;\ldots \;h(N_{D}-1,D)\right]}^{T}$ are the kernel coefficients of the nonlinear model whose original value is an P-dimensional zero column vector.

The nonlinear model kernel u(n) is a P-dimensional column vector produced by large signals and denoted as:

(24) $$\begin{array}{cc} u(n)= & \left[L_{1}(n)\;L_{1}(n-1)\;\ldots \;L_{1}(n-N_{1}+1\right)\\ & \;{\left(L_{1}(n)\right)}^{2}\;{\left(L_{1}(n-1)\right)}^{2}\;\ldots \;{\left(L_{1}(n-N_{2}+1)\right)}^{2}\\ & \;........\\ & {\left(L_{1}(n)\right)}^{D}\;{\left(L_{1}(n-1)\right)}^{D}\;\ldots \;{\left(L_{1}(n-N_{D}+1)\right)}^{D}{]}^{T} \end{array}$$

and:

(25) $$J(\omega )={\left[e(n)\right]}^{T}e(n)=\sum_{k=1}^{N}{\left[e(k,\omega )\right]}^{2}$$

where, e(k,ω) stands for the kth point e(n); $e(n)={\left[e(1,\omega ),e(2,\omega )\ldots e(N,\omega )\right]}^{T}$ (1≤k≤N) is N×1; J(ω) means the power of N points e(n).

(26) $$e(k,\omega )=S_{1}(n-k+1)-u^{T}(n-k+1)\omega (n)$$

where, $S_{1}(n-k+1)$ and u(n−k+1) signify small signals and nonlinear model kernel of the kth point in the first channel, respectively.

It can be obtained from Equations (25) and (26):

(27) $$\begin{array}{ccc} J(\omega ) & = & \sum_{k=1}^{N}{\left[e(k,\omega )\right]}^{2}=\sum_{k=1}^{N}{\left[e(k,\omega )\right]}^{T}e(k,\omega )\\ & = & \sum_{k=1}^{N}{\left[S_{1}(n-k+1)-u^{T}(n-k+1)\omega \right]}^{T}[S_{1}(n-k+1)-u^{T}(n-k+1)\omega (n)]\\ & = & \sum_{k=1}^{N}S_{1}{}^{T}(n-k+1)S_{1}(n-k+1)-\sum_{k=1}^{N}{S_{1}}^{T}(n-k+1)u^{T}(n-k+1)\omega (n)\\ & & -\sum_{k=1}^{N}\omega^{T}u(n-k+1)S_{1}(n-k+1)+\sum_{k=1}^{N}\omega^{T}u(n-k+1)u^{T}(n-k+1)\omega (n) \end{array}$$

The objective function is to minimize J(ω), therefore, the authors compute the gradient of J(ω) with respect to ω(n):

(28) $$\begin{array}{l} -\sum_{k=1}^{N}{\left[{S_{1}}^{T}(n-k+1)u^{T}(n-k+1)\right]}^{T}-\sum_{k=1}^{N}u(n-k+1)S_{1}(n-k+1)\\ +2[\sum_{k=1}^{N}u(n-k+1)u^{T}(n-k+1)]\omega_{opt}(n)=0 \end{array}$$

Continuing to earn Equation (29):

(29) $$K(n)K^{T}(n)\omega_{opt}(n)=K(n)Y(n)$$

where $Y(n)={\left[S_{1}(n),S_{1}(n-1),S_{1}(n-2),\ldots S_{1}(n-N+1)\right]}^{T}$ is N×1, K(n)=[u(n),u(n−1),u(n−2),…u(n−N+1)] is P×N and the kernel vector of the nonlinear behavior model $\omega_{opt}(n)$ means the solution of the objective function.

Simplifying Equation (29) to earn Equation (30):

(30) $$KK^{T}\omega_{opt}=KY$$

where, K(n), $\omega_{opt}(n)$ and Y(n) is abbreviated to K, $\omega_{opt}$ and Y, respectively.

Last, based on the proposed compensation strategy, the authors acquire:

(31) $$\begin{array}{cc} z_{i}=y_{i}-{U_{i}}^{T}\omega_{opt} & 1\leq i\leq m \end{array}$$

where $U_{i}$ (the facilitation form of $U_{i}(n)$) denotes the pipeline model kernel of the ith channel, while $U_{i}(n)$ is expressed as:

(32) $$\begin{array}{cc} U_{i}(n)= & \left[y_{i}(n)\;y_{i}(n-1)\ldots y_{i}(n-N_{1}+1\right)\\ & {\left(y_{i}(n)\right)}^{2}\;{\left(y_{i}(n-1)\right)}^{2}\ldots {\left(y_{i}(n-N_{2}+1)\right)}^{2}\\ & ........\\ & {\left(y_{i}(n)\right)}^{D}\;{\left(y_{i}(n-1)\right)}^{D}\ldots {\left(y_{i}(n-N_{D}+1)\right)}^{D}{]}^{T} \end{array}$$

The specific procedures to resolve Equation (30) are discussed as the following:

The orders of the Hammerstein series are set from 1 to 5 in Equation (30). Due to a very great power difference among various order terms, obviously $KK^{T}$ is considered as an ill-conditioned matrix. Consequently, the calculation of the model kernel vector can be converted into that of ill-conditioned linear equations. Here, the IWI method is present to calculate the ill-conditioned equations. Representing a given iteration l, the iterative formula is:

(33) $$\begin{array}{cc} \left(A+\lambda I\right)X^{\left(l+1\right)}=b+\lambda X^{\left(l\right)} & \left(A=KK^{T}\;b=KY\right) \end{array}$$

where λ represents a non-zero constant, l expresses the iteration index, $X^{\left(0\right)}$ is the initial value of the equations solution, $X^{\left(l\right)}$ is the approximate estimate at lth iteration.

**Figure 4:**
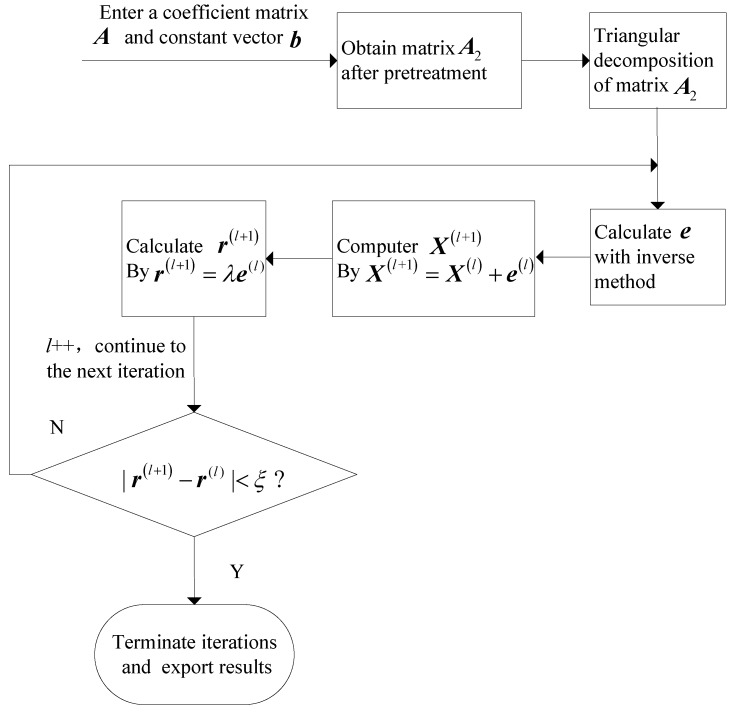
Flows of adaptive iteration for Hammerstein kernel vector.

Figure 4 illustrates the concrete steps of IWI algorithm, where $A_{2}=A+\lambda I$, $r^{\left(l\right)}$ and $e^{\left(l\right)}$ indicate the margin and error at iteration l, respectively, l++ means l increasing by 1 in every iteration.

### 3.2. SRA-TFC Method

The key point of the proposed nonlinear blind compensation method is to gain large signals $L_{1}(n)$ and small signals $S_{1}(n)$ blindly and precisely. The following are the details regarding the proposed blind signal separation method.

**Figure 5:**
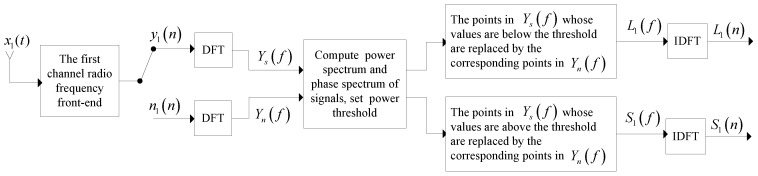
Proposed blind signal separation method structure.

According to Figure 5, the SRA-TFC algorithm can be divided into the following four steps:

1. The time domain distorted signal $y_{1}\left(n\right)$ and pure noise $n_{1}\left(n\right)$ from the first channel RF front-end needs to be extracted.
2. $y_{1}\left(n\right)$ and $n_{1}\left(n\right)$ are both translated into the corresponding signal and noise in frequency-domain via the Discrete Fourier transform (DFT) technique and denoted as $Y_{s}\left(f\right)$ and $Y_{n}\left(f\right)$, respectively.
3. Each point of $Y_{s}\left(f\right)$ is compared with the setting power spectrum threshold. Next, frequency-domain large signals $L_{1}\left(f\right)$ are obtained with the method that the points in $Y_{s}\left(f\right)$, whose values are below the threshold, are replaced by the corresponding points in $Y_{n}\left(f\right)$, whereas the others remain. Conversely, frequency-domain small signals, $S_{1}\left(f\right)$ is gained by the replaced points in $Y_{s}\left(f\right)$ whose values are above the threshold.
4. By the IDFT technique, the authors turn $L_{1}\left(f\right)$ and $S_{1}\left(f\right)$ to time-domain large signals, $L_{1}(n)$ and time-domain small signals, $S_{1}(n)$, separately.

### 3.3. Comparisons between SRA-TFC Method and Traditional SVD-Based Method

To demonstrate the advantages of the proposed method, the authors compare the SRA-TFC signal separation method to the traditional SVD-based signal separation method in terms of the compensation performance and the computational complexity.

#### 3.3.1. Comparison of Compensation Results

**Figure 6:**
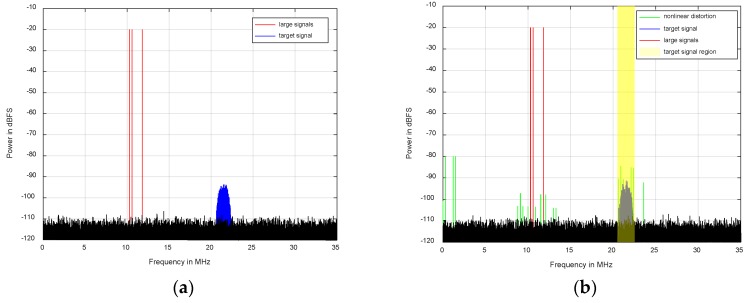
Classic simulation results of a distorted spectrum: (**a**) original signal; (**b**) distorted signal.

**Figure 7:**
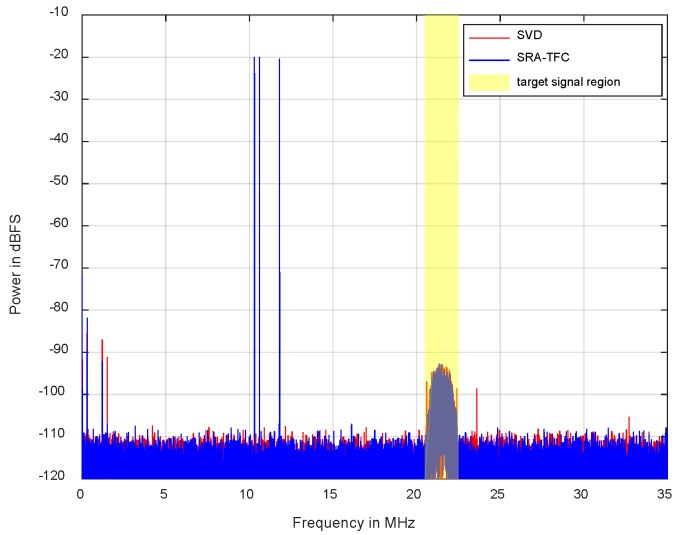
Nonlinearity mitigation results of typical simulation signals for two blind signal separation algorithms.

The simulation compensation results are provided to testify the advantages of the SRA-TFC signal separation method. To focus on the comparison of these two signal separation methods, the parameters of any one of the channels are adaptively extracted to alleviate the nonlinearity of this channel. It is noteworthy that the number of the sampling points for power spectrum analysis is 65,536 in Figure 6 and Figure 7.

Figure 6a depicts that the original simulation signals cover the equal-amplitude three-tone strong sinusoidal wave signal (large signals) and the weak 16-QAM (quadrature amplitude modulation) modulation signal (target signal). The frequencies of large signals are 10.3 MHz, 10.6 MHz and 11.8 MHz and the center frequency and bandwidth of the weak modulation signal is 21.5 MHz and 1 MHz, respectively. Moreover, the power spectrum difference between large signals and the target signal is about 75 dB and the sample rate is 70 MHz. A Hammerstein model with *D* = 5 and *N_d_* = [2, 2, 2, 2, 2] is adopted. As shown in Figure 6b, the signal and carrier frequencies are chosen arbitrarily, the only condition being that some large nonlinear distortion components should be in the target signal region to dramatically reduce the SINR of the target signal.

Regarding Figure 7, the blue spectrum illustrates the compensation performance when the SRA-TFC signal separation method is employed, while the red spectrum indicates the mitigation result in the application of the SVD-based signal separation method. While both signal separation methods perform adequately, the proposed method demonstrates slightly better overall mitigation performance.

**Figure 8:**
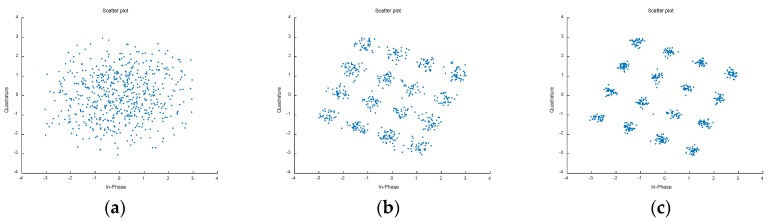
The constellations of the 16-QAM (quadrature amplitude modulation) signals: (**a**) distorted weak 16-QAM; (**b**) weak 16-QAM after mitigation by singular value decomposition (SVD)-based signal separation method; (**c**) weak 16-QAM after mitigation by the proposed signal separation method.

Furthermore, as depicted in Figure 6b, the SINR of the weak 16-QAM signal is dramatically reduced due to the large disturbance stemming from the large signals. Moreover, after the processes of frequency selection, filtering and demodulation for the weak 16-QAM signal, its constellation is very messy, as shown in Figure 8a. However, as illustrated in Figure 8b,c, benefiting from the proposed algorithm, the nonlinear distortion in the target signal region is obviously depressed. Finally, a regularly-distributed constellation is obtained. Moreover, compared to Figure 8b, the constellation in Figure 8c is more regular because the novel algorithm has better compensation performance in the target signal region.

This experiment illustrates that the nonlinear blind compensation method can improve the SINR of the weak 16-QAM signal but not change the weak signal itself, which is a crucial capacity of the parameter estimation for target signals.

#### 3.3.2. Comparison between Computational Complexities

The computational complexities of the proposed method and the traditional SVD-based algorithm are discussed in this section. Let the data length N=M×M where M is applied to be the measurement base of complexity.

Separating large signals and small signals from the one-channel output signal, the required calculations of the traditional SVD-based algorithm include $O\left(M^{3}\right)$ times addition, $O\left(M^{3}\right)$ iterations of multiplication, $O\left(M^{2}\right)$ iterations of division and $O\left(M^{2}\right)$ times square-root, to the knowledge of Reference [27].

According to the procedures described in Section 3.2, the required computational complexity of the proposed method is displayed as the following:

1. N points $y_{1}\left(n\right)$ and N points $n_{1}\left(n\right)$ are first extracted and then separately converted into $Y_{s}\left(f\right)$ and $Y_{n}\left(f\right)$ via DFT. The N points DFT requires both NlogN times of multiplication and NlogN times of addition. Therefore, the required calculations to acquire $Y_{s}\left(f\right)$ and $Y_{n}\left(f\right)$ are 2NlogN iterations of multiplication and addition.
2. It requires 2N times multiplication and N times addition to calculate the power spectral density of $Y_{s}\left(f\right)$. Moreover, with the application of above threshold detection method, it needs 2N iterations of addition to achieve $L_{1}\left(f\right)$ and $S_{1}\left(f\right)$.
3. The authors separately convert $L_{1}\left(f\right)$ and $S_{1}\left(f\right)$ into $L_{1}(n)$ and $S_{1}(n)$ by IDFT. This step requires 2NlogN iterations of multiplication and addition.

According to Steps 1–3, the proposed signal separation method requires $O\left(N\right)=O\left(M^{2}\right)$ iterations of multiplication and addition. The authors summarize the computational complexities of the two methods in Table 2. It can be observed that the computational complexity of the proposed method is O(M) times less than that of SVD-based method. 

Compared to the traditional SVD-based algorithm, in summary, the proposed method has much lower computational loads but achieves a slightly better mitigation performance.

## 4. Experimental Results and Analysis

An experimental verification platform is built to validate the performance of the proposed algorithm. Moreover, the real-world experimental results show the impact of the nonlinearity mitigation of the array receiver on the source localization accuracy of target signals and evaluate the performance of the proposed algorithm.

### 4.1. Nonlinearity Mitigation Performance for ASP System

Relative to the other array geometries, uniform circular array (UCA) has several advantages such as its 360° azimuth angle coverage and additional elevation angle information [34,35]. Additionally, it has wide application in 2-D DOA estimation. Therefore, according to Figure 1, the shortwave array signal used for testing is from an actual UCA with sixteen elements. The experimental array receiver is a 16-channel receiver and its synchronous sampling rate is $f_{s}=70\;\mathrm{MHz}$.

**Figure 9:**
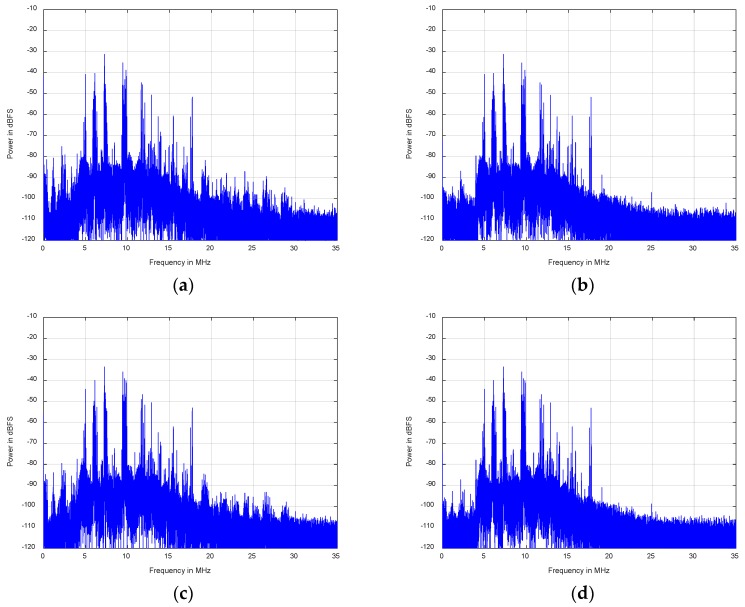
Power spectrum comparison between the original and post-compensation of array signal versus different channel: (**a**) the original result in the first channel; (**b**) post-compensation result in the first channel; (**c**) the original result in the second channel; (**d**) post-compensation result in the second channel.

The model parameters of the first channel are extracted and then to alleviate the nonlinear distortion components of all channels in this experiment. Additionally, the nonlinear inverse behavior model for all channels’ RF front-ends is employed by a Hammerstein model with *D* = 5 and *N_d_* = [3, 3, 3, 3, 3]. Figure 9a,b show the power spectrum of the original and post-compensation array signal in the first channel. Since the input array signals of all channels are similar and their compensation results are also similar, it is only necessary to show the power spectrum of the signal before and after compensation in other channels such as the second one, which are shown in Figure 9c,d.

Moreover, Figure 9a,b illustrates that the SFDR of the first channel has a 10–20 dB improvement in the whole band by carrying out the presented compensation method. Meanwhile, the same conclusion of the second channel from Figure 9c,d can be drawn. Therefore, it can be deduced that the SFDR of the entire array receiver in in the full band rises by 10–20 dB through the implementation of the proposed compensation method.

**Figure 10:**
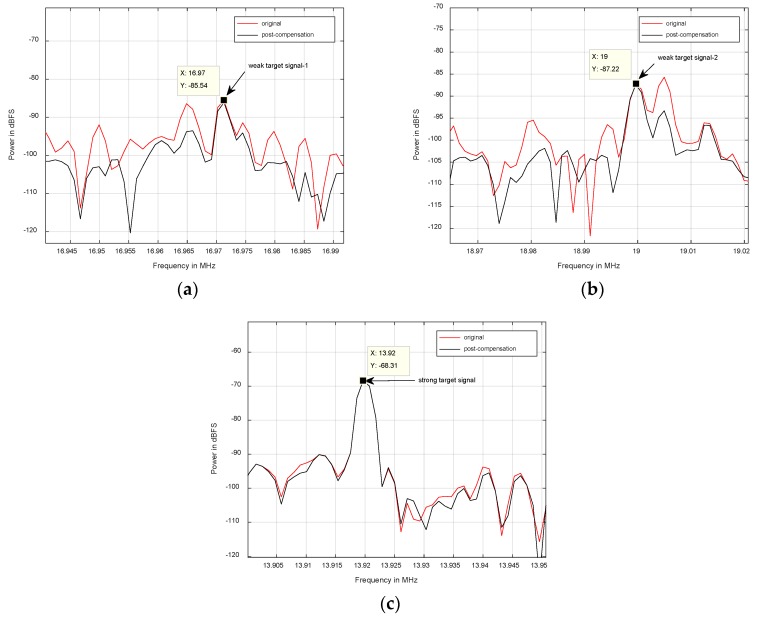
Power spectrum comparison between the original and post-compensation of three target signals in the first channel: (**a**) weak target signal-1; (**b**) weak target signal-2; (**c**) strong target signal.

Meanwhile, three target signals (two weak ones and a strong one) are chosen to dissect the effect of the nonlinearity mitigation of array signal processing system on the 2-D DOA estimation performance of array signal. Displayed in Figure 10a–c (the local power spectrum graphs of the first channel), there are three array signals of which center frequencies are 16.97 MHz, 19 MHz and 13.92 MHz, respectively. Meanwhile, the powers of the two array signals in Figure 10a,b are both almost 20 dB lower than the power of the array signal in Figure 10c. Therefore, the three targets are named weak target signal-1, weak target signal-2 and strong target signal, respectively.

Figure 10a,b illustrate that the two weak array signals are both seriously interfered by unwanted in-band nonlinearity and that the SINRs of the two signals are below 0 dB. However, after compensation, the SINRs of the two signals are both increased by approximately 10 dB. Therefore, the two array signals are the typical examples the authors research.

Contrarily, as shown in Figure 10c, the power spectrums of the strong array signal before and after compensation are almost similar, owing to a total lack of the nonlinear distortion components. Additionally, the SINRs of the original and post-compensation signals are approximately 20 dB.

### 4.2. 2-D DOA Performances of Target Signals

**Figure 11:**
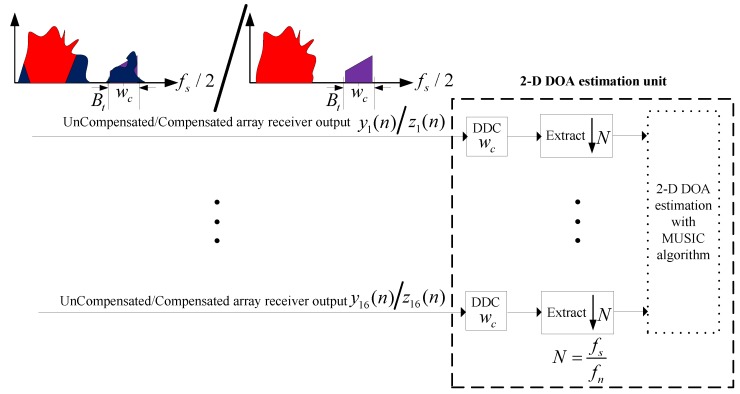
Two-dimensional direction-of-arrival (2-D DOA) estimation structure.

The direction-finding performances of the above-mentioned array signals before compensation versus after compensation are discussed. Figure 11 shows the 16-channel array receiver output before compensation is processed through the frequency selection and channelization before their 2-D DOAs (elevation angle α and azimuth angle β) are estimated as is the compensated array receiver output where, the channelized sampling rate and bandwidth are $f_{n}$ = 10 KHz and 8 KHz, respectively. Moreover, the authors employ the MUSIC algorithm to estimate their 2-D DOAs of array signal before and after compensation. Ultimately, the authors provide explicit comparisons about these two estimation results. 

The array receiver output with 118 s in succession is used to estimate the 2-D DOA parameters based on the MUSIC algorithm. Where one-second array signal data is estimated at a time. Thereupon, one hundred and eighteen independent Monte Carlo trials are performed for the following experiments. Moreover, the root mean square error (RMSE) is used for the performance measure. The RMSE of elevation and azimuth DOA (RMSE(α) and RMSE(β)) are defined as:

(34) $$RMSE(\alpha )=\sqrt{\frac{1}{118}\sum_{t=1}^{118}{\left(\alpha_{t}-\tilde{\alpha }\right)}^{2}}$$

(35) $$RMSE(\beta )=\sqrt{\frac{1}{118}\sum_{t=1}^{118}{\left(\beta_{t}-\tilde{\beta }\right)}^{2}}$$

where $\alpha_{t}$ and $\beta_{t}$ mean the estimation of the elevation angle and azimuth angle at the tth second (the tth Monte Carlo trial), separately, while $\tilde{\alpha }$ and $\tilde{\beta }$ stand for the corresponding average ones.

#### 4.2.1. 2-D DOA Performance in the Case of Weak Target Signals

These two experiments are to compare the 2-D DOA estimation results of weak target signals before and after compensation.

**Figure 12:**
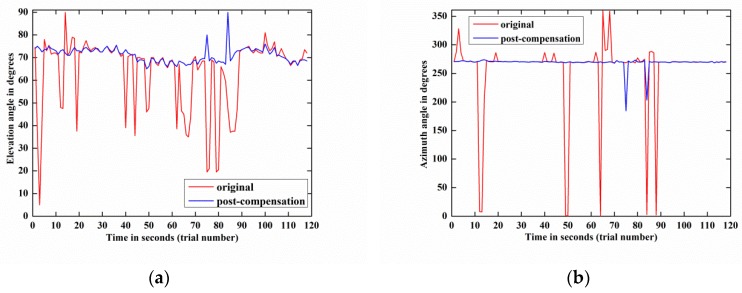
2-D DOA estimation performance comparison between the original and post-compensation of weak target signal-1 by 118 trials: (**a**) Elevation angle; (**b**) Azimuth angle.

**Figure 13:**
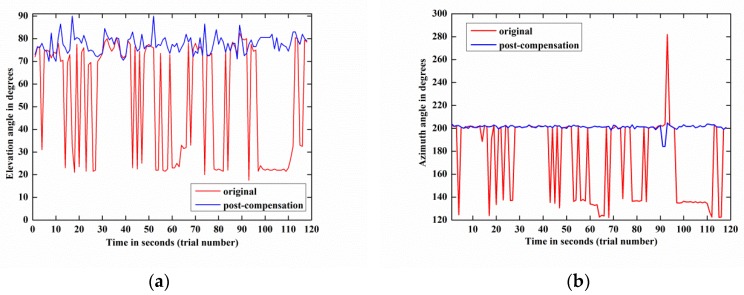
2-D DOA estimation performance comparison between the original and post-compensation of weak target signal-2 by 118 trials: (**a**) Elevation angle; (**b**) Azimuth angle.

**Figure 14:**
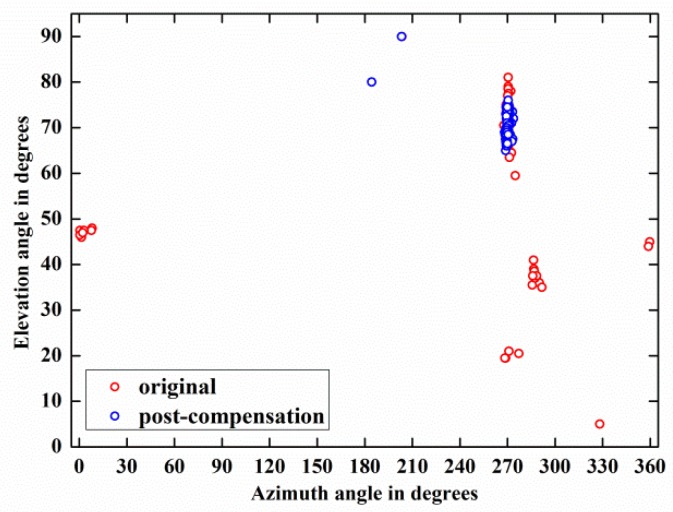
Azimuth-elevation angle estimation results comparison between weak target signal-1 before and after compensation.

**Figure 15:**
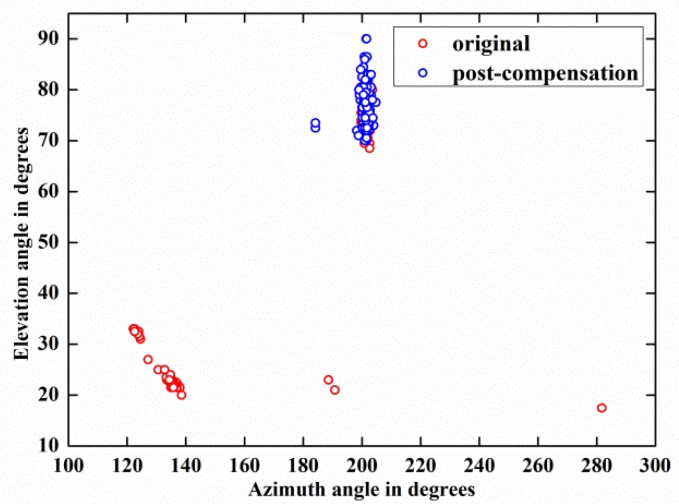
Azimuth-elevation angle estimation results comparison between weak target signal-2 before and after compensation.

Figure 12 depicts the DOA estimation performances of weak target signal-1 before and after compensation by 118 trials and demonstrates that the estimation result of the post-compensation signal is much more accurate than that of the original signal. Meanwhile, the same phenomenon also happens on weak target signal-2, as shown in Figure 13. These phenomena are observed more directly from the azimuth-elevation scatter figures, Figure 14 and Figure 15.

Moreover, by carrying out the compensation algorithm, the RMSE(α) of weak target signal-1 is reduced from 15.62° to 3.31° and the RMSE(β) is decreased from 65.81° to 10.03°. Concurrently, for weak target signal-2, its RMSE(α) and RMSE(β) are reduced from 25.66° to 3.94° and from 34.98° to 2.48° by employing the blind compensation algorithm.

The above two experimental results indicate that the proposed algorithm can dramatically increase the 2-D DOA estimation accuracy of the weak target signals.

#### 4.2.2. 2-D DOA Performance in the Case of Strong Target Signal

Another crucial concern to the authors is the azimuth-elevation direction finding performance analysis of strong array signal before compensation versus after compensation.

**Figure 16:**
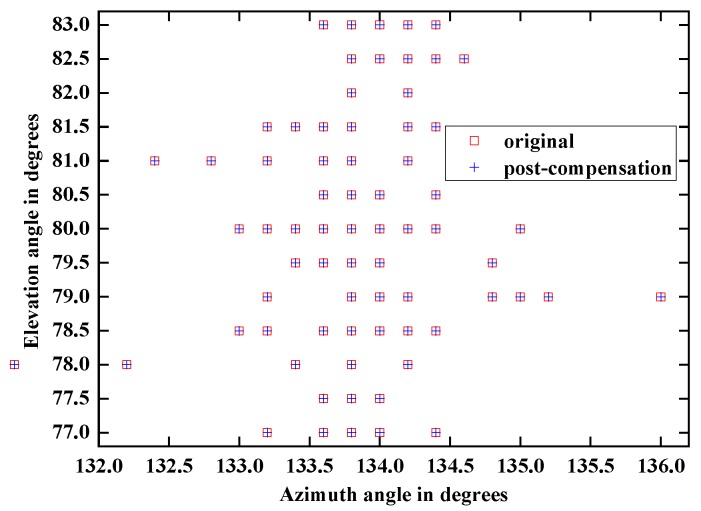
Azimuth-elevation angle estimation results comparison between strong target signal before and after compensation.

It can be seen in Figure 16 that the azimuth-elevation angle estimation results of strong target signal before and after compensation are identical. The experimental result suggests that the proposed method just eliminates the nonlinearity without affecting the strong signal and its parametric estimation accuracy.

## 5. Conclusions

Rather than developing different parametric estimation methods, the authors investigated a nonlinear blind compensation technique in array signal processing. A novel blind compensation algorithm was presented to improve the SFDR of an array receiver and to obtain remarkable improvements of the parameters estimation performances for weak target signals such as their 2-D DOAs employing the MUSIC algorithm. These purposes have already been verified by the repeated experiments on a real-world array signal from a UCA. The main advantages of the proposed algorithm are summed up in the following points:

1. During the blind compensation process, the parameters of the identification module and the compensation module are totally independent of each other, which can improve the efficiency of array signal processing and increase its dependability.
2. The suggested algorithm accomplished in pure time-domain saves a great deal of the system hardware scale. In addition, the proposed algorithm is only necessary to set power threshold rather than multi-stopband/multi-passband digital filters with extremely high performance of constant change, which is apparently more flexible and convenient to handle the situation of multiple signals with different power levels or wide ranges of bandwidth.
3. The blind compensation strategy for multi-channel RF front-ends designed is that the model parameters of any one of the channels are extracted to mitigate the nonlinear distortion components of all channels synchronously. It has the advantages of reducing a mountain of computational loads and avoiding the inconsistency of the array compensation performance caused by the iterative computation error, especially the phase disturbance of the array signal after compensation.

In conclusion, the proposed algorithm can improve the SFDR of the array receiver, achieving high parameters estimation accuracy of array signal with low computational complexity. It is predicted to have wide and promising applications in array signal processing.

## Figures and Tables

**Table 1:** The maximum and minimum absolute values of *PE_i_* versus different frequency points.

Signal Frequency (MHz)	Computational Complexity	
	Maximum |*PE_i_*| (dB)	Minimum |*PE_i_*| (dB)
5.02	*PE*_14_ = 1.166	*PE*_5_ = 0.143
13.92	*PE*_10_ = 0.583	*PE*_3_ = 0.008
16.97	*PE*_5_ = 0.582	*PE*_6_ = 0.022
19	*PE*_10_ = 0.697	*PE*_3_ = 0.029
21.6	*PE*_10_ = 0.318	*PE*_2_ = 0.019
28.02	*PE*_5_ = 0.976	*PE*_10_ = 0.04

**Table 2:** Comparisons of the computational complexities between two signal separation methods.

Signal Separation Method	Computational Complexity			
	Addition	Multiplication	Division	Square-Root
traditional SVD-based method	$O\left(M^{3}\right)$	$O\left(M^{3}\right)$	$O\left(M^{2}\right)$	O(M)
SRA-TFC method	$O\left(M^{2}\right)$	$O\left(M^{2}\right)$	without	without
